# Feasibility and Outcomes of “No-Look No-Touch” Laparoscopic Radical Trachelectomy for Early-Stage Cervical Cancer

**DOI:** 10.3390/jcm10184154

**Published:** 2021-09-15

**Authors:** Hiroyuki Kanao, Yoichi Aoki, Atsushi Fusegi, Makiko Omi, Hidetaka Nomura, Terumi Tanigawa, Sanshiro Okamoto, Tomoko Kurita, Sachiho Netsu, Kohei Omatsu, Mayu Yunokawa

**Affiliations:** 1 Department of Gynecologic Oncology, Cancer Institute Hospital, Tokyo 135-8550, Japan; yoichi.aoki@jfcr.or.jp (Y.A.); atsushi.fusegi@jfcr.or.jp (A.F.); makiko.omi@jfcr.or.jp (M.O.); hidetaka.nomura@jfcr.or.jp (H.N.); terumi.tanigawa@jfcr.or.jp (T.T.); sanshiro.okamoto@jfcr.or.jp (S.O.); sachiho.netsu@jfcr.or.jp (S.N.); kohei.omatsu@jfcr.or.jp (K.O.); mayu.yunokawa@jfcr.or.jp (M.Y.); 2 Department of Gynecologic Oncology, Hospital of the University of Occupational and Environmental Health, Fukuoka 807-8556, Japan; tomoko.kurita@jfcr.or.jp

**Keywords:** cervical cancer, laparoscopy, radical trachelectomy, uterine artery, survival, pregnancy, delivery

## Abstract

Intraoperative tumor manipulation and dissemination may compromise the survival of women with early-stage cervical cancer who undergo laparoscopic surgery. This study aimed to examine survival and obstetrical outcomes related to laparoscopic radical trachelectomy (LRT) with a “no-look no-touch” technique in 40 women. This technique incorporates five measures to prevent tumor spillage and damage to the uterine artery perfusion. Five LRTs were aborted because of positive nodes or positive surgical margins. Compared with those of type III laparoscopic radical hysterectomy, the surgical outcomes of LRT in 35 patients were acceptable: operative time (380 min), estimated blood loss (140 mL), length of hospital stay (15 days), and lengths of excised parametrium and vagina. During follow-up (median, 41.3 months), the 5-year disease-free survival and overall survival were 95.0% (95% CI: 69.5–99.3%) and 100%, respectively. Of the nine patients (26%) who attempted pregnancy, seven conceived (nine pregnancies, 76%). Eight were delivered by term cesarean section, while one was miscarried in the first trimester. Our study suggests that the no-look no-touch technique may be effective in reducing the risk of recurrence and improving obstetrical outcomes during LRT for early-stage cervical cancer.

## 1. Introduction

Radical trachelectomy, first reported by Dargent in 1994 [1], is now considered a treatment option for patients with early-stage cervical cancer who desire fertility preservation. With the rapidly growing availability and technical feasibility of laparoscopic surgery, laparoscopic radical trachelectomy (LRT) has been widely accepted [2]. Previous reports demonstrated the technical feasibility and advantages of LRT in terms of reduced blood loss and shorter hospital stay [3], and a systematic review of 47 articles that reported 2566 patients who underwent radical trachelectomy demonstrated equivalent oncologic outcomes between abdominal, vaginal, and laparoscopic approaches [4]. Thus, LRT has recently become an appropriate treatment choice for patients with cervical cancer with fertility preservation. 

In 2018, the Laparoscopic Approach to Cervical Cancer (LACC) trial, which is a phase 3, multicenter, randomized study, revealed that laparoscopic or robotic radical hysterectomy was associated with poor prognosis compared with open abdominal radical hysterectomy, with the risks of recurrence and death being four and six times higher, respectively [5]. These unexpected results are supposed to be induced by cancer cell spillage under CO_2_ circulation [6]. 

Peritoneal dissemination, which was caused by cancer cell spillage during the surgery, was a matter of a great concern, when laparoscopy was first introduced for the resection surgery of rectal cancer [7]. Tumor perforation, excessive manipulation of the tumor, and tumor spread via pneumoperitoneal CO_2_ are thought to cause peritoneal dissemination [8,9,10,11]. Thus, cancer cell spillage resulting in peritoneal dissemination is thought to be preventable in many cases [12].

Laparoscopic radical hysterectomy (LRH) includes several procedural steps that can lead to cancer cell spillage. Insertion of a uterine manipulator can lead to tumor perforation, squeezing of the uterine cervix can result in tumor spillage, and intraperitoneal colpotomy can cause tumor exposure to the circulating CO_2_. Previously, we incorporated a specific procedure to prevent cancer cell spillage into the surgical field during LRH and demonstrated its technical feasibility and equivalent oncologic outcomes in comparison with abdominal radical hysterectomy [13]. 

When discussing any surgical interventions for cervical cancer, we should always consider the results of the LACC trial. Similar to LRH, LRT also includes several procedural steps that can lead to intraoperative tumor spillage. To prevent repeating the history of the LACC trial, we believe that protective maneuvers of cancer cell spillage should be mandatory in LRT, which requires the use of additional protection techniques during trachelectomy.

One of the important issues in radical trachelectomy is the preservation of the uterine arteries. Theoretically, sacrifice of a uterine artery may pose a potentially adverse impact on future fertility; however, this is not clear due to limited cases [14,15]. 

Preservation of the uterine artery during LRT is technically challenging. Tang et al. reported that 87.2% of anatomically preserved uterine arteries during radical trachelectomy led to occlusion due to thermal injury or traumatic handling [16], and the utility of ICG fluorescence angiography was demonstrated to provide real-time evaluation of uterine perfusion after radical trachelectomy [17].

With the goal of ensuring oncologic and obstetrical outcomes, we performed no-look, no-touch LRT, which incorporated specific procedures to prevent cancer cell spillage into the peritoneal cavity and allowed for uterine artery preservation with real-time evaluation. 

The objective of this study was to examine the technical feasibility as well as the oncological and obstetrical outcomes related to no-look no-touch LRT for early-stage cervical cancer. 

## 2. Patients and Methods

### 2.1. Criteria for Patient Selection and Conversion to Laparoscopic Radical Hysterectomy (LRH)

Patients with stage 1A2–2A1 cervical cancer desiring fertility preservation were candidates for our no-look, no-touch laparoscopic radical trachelectomy (NLNT-LRT) if they met the following criteria:

The cervical cancer was estimated on preoperative imaging to be less than 20 mm without any extracervical involvement or distant metastatic lesion.

Preoperative MRI ruled out upper endocervical involvement and ensured a residual cervical length of >20 mm (i.e., the length between the internal os and the tumor was estimated to be more than 20 mm).

During the NLNT-LRT, cytologic examination of ascitic fluid was performed, and sentinel lymph nodes and/or swelling nodes, as well as margins of trachelectomy, were submitted for frozen sections. When any of them were positive for cancer, the patients underwent conversion to LRH.

This study included patients with the clinical FIGO stage 1A2–2A1 cervical cancer who underwent NLNT-LRT between 2015 and 2020 at the Cancer Institute Hospital (*n* = 40). In all cases, the type III nerve-sparing technique and pelvic lymphadenectomy were performed by the same experienced board-certified laparoscopic surgeon (H.K) in the same manner, incorporating specific measures designed to prevent cancer cell spillage and preserve uterine artery perfusion. This study was approved by the Institutional Review Board of our facility.

### 2.2. Specific Measures to Prevent Tumor Spillage and Preserve Uterine Artery Perfusion during NLNT-LRT

Our NLNT-LRT procedure includes five specific measures adopted to prevent tumor spillage and preserve uterine artery perfusion: (1) creation of a vaginal cuff, (2) manipulation of the uterus without insertion of a uterine manipulator, (3) minimal handling of the uterine cervix and uterine artery, (4) real-time evaluation of uterine artery perfusion using indocyanine green (ICG) intraoperative angiography, and (5) extracorporeal trachelectomy (Supplementary Video S1).

A step-by-step procedure for the no-look no-touch technique is described below. The fundamental concept of this technique is that the tumor should not be exposed under CO_2_ circulation (no-look technique), and direct manipulation of the tumor and the uterine artery should be avoided (no-touch technique) during LRT, as these may cause tumor spillage into the surgical field and may damage uterine artery perfusion. 

#### 2.2.1. Creation of a Vaginal Cuff

Twelve sutures were placed circumferentially, approximately 2 cm from the tumor, and the sutures were pulled to reveal the incision line. The vaginal mucosa was then incised circumferentially with a monopolar device, and a vaginal cuff was completed by closing the incised vaginal mucosa with a double layer of continuous sutures. After the creation of a vaginal cuff, the tumor was not exposed during the surgery. 

#### 2.2.2. Manipulation of the Uterus without Insertion of a Uterine Manipulator

A 5-mm extra-long trocar (150 mm in length) was inserted at the posterior incision line of the vaginal mucosa and forceps were introduced through this port. A 1-0 Vicryl suture was placed around the uterine body, and the forceps were used to push the suture to mobilize the uterus without using a uterine manipulator.

#### 2.2.3. Minimal Handling of the Uterine Cervix and Uterine Artery

In some cases, as in obese patients, the operative view is poor, and the uterine cervix must be squeezed both medially and laterally to expose the surgical field. This direct handling of the cervix may result in cancer cell spillage. Our suspension technique seems to be useful for minimizing direct handling of the uterine cervix.

Avascular space between the mesorectal fascia and presacral fascia was dissected and developed down to the levator ani muscle. Then, the rectum was mobilized and the inferior hypogastric nerves and pelvic nerve plexus were identified and preserved. To expose the right-side parametrium, the rectum was lifted toward the opposite (left) side, and the umbilical ligament was lifted toward the right side. This suspension technique could avoid the need to squeeze the uterine cervix to create a sufficient surgical field. 

The uterine artery was gently exposed and encircled by an 8-cm vessel tape. It was manipulated by pulling and pushing the vessel tape, with direct handling of the uterine artery avoided. Thermal spread injury was also a great concern, therefore, minimal use of energy devices around the uterine artery was essential. A monopolar device should be noted as having the greatest degree of thermal spread, only a pure-cut current with a low voltage (20 W) was used when a monopolar device was applied around the uterine artery.

#### 2.2.4. Real-Time Evaluation of Uterine Artery Perfusion with Indocyanine Green (ICG) Intraoperative Angiography

During LRT, 2 mL diluted ICG (2.5 mg/mL) was injected intravenously and uterine artery blood flow was evaluated using the IMAGE 1S camera system (KARL STORZ SE & Co. KG; Tuttlingen, Germany).

#### 2.2.5. Extracorporeal Trachelectomy

After colpotomy, the uterus was attached to the uterine artery as well as to the infundibulopelvic and round ligaments. A 7 cm lower abdominal transverse incision was made, and the uterine cervix was extracted from the pelvic cavity. The anatomical position of the internal os was identified by ultrasonography, and trachelectomy was conducted 10 mm below the internal os; that is, a residual cervical length of 10 mm. After placement of a 2–0 prorin-permanent cerclage, anastomosis between the cervix and vagina was performed laparoscopically.

### 2.3. Postoperative Management and Surveillance

After NLNT-LRT, patients with a risk for recurrence (namely, with an intermediate risk factor of lymphovascular space involvement, and/or >50% myometrial invasion) received adjuvant chemotherapy as described in the JSGO guidelines [18]. Upon completion of treatment, patients underwent a follow-up examination every 3 months and computed tomography (CT) or positron emission tomography (PET CT) with a 12-month interval to evaluate tumor recurrence. In cases of subjective symptoms or clinical signs of tumor recurrence, a comprehensive diagnostic workup was initiated.

At 6 months after the completion of treatment, the patients were allowed to conceive.

### 2.4. Study Variables

The patients’ clinical characteristics and operative outcomes were retrieved from the hospital records, which included information on the following: the age, body mass index (BMI); parity; history of conization; operation time; blood loss volume; lengths of the excised parametrium, vaginal cuff, and residual cervix; time to recovery of normal bladder function; length of hospital stay (days); intraoperative and/or postoperative complications; tumor diameter; histologic type (squamous cell carcinoma (SCC) versus non-SCC); status of the surgical margin; surgical-pathological tumor stage; type of adjuvant therapy; and disease-free survival (DFS) and overall survival (OS). The obstetrical outcomes were also retrieved from the hospital records, including the number of patients attempting pregnancy, the number of patients who achieved pregnancy, and the outcomes of pregnancy. 

Complications were defined as the occurrence of any event during or after surgery that required further surgical procedures, interventional radiotherapy, or rehabilitation therapy. Normal bladder function was defined as a post-void residual urine volume of <50 mL. The length of the parametrium and the vaginal cuff were measured linearly from their attachment to the uterine cervix. The length of the residual cervix was measured usingultrasonography one month after LRT. DFS was defined as the time between the surgery and the time of initial recurrence or death from cervical cancer, and OS was defined as the time between the surgery and the time of death from any cause. Patients known to be disease-free or alive at their last contact date were censored. 

### 2.5. Statistical Analysis

A standard descriptive analysis was performed. Clinicopathological variables were expressed as the median and interquartile range (IQR) or as the number and percentage of patients. Intra-group differences in continuous variables were analyzed using an independent sample t-test or Mann-Whitney U test depending on normality. DFS and OS were constructed using the Kaplan-Meier method. All statistical analyses were performed using SAS software, version 9.4 (SAS institute Inc., Cary, NC, USA) and EZR [19], and statistical significance was set at *p* < 0.05.

## 3. Results

The clinical characteristics of the study patients are summarized in Table 1. The median age was 34 years (range: 30–37 years), median BMI was 20.1 kg/m^2^ (range: 18.2–21.9), and almost all were nulliparous (98%). The median tumor size was 13.5 mm (range: 8.1–20 mm), and most of the tumors were stage 1b1 (*n* = 29, 73%). Approximately 43% of patients (*n* = 17) underwent diagnostic conization prior to LRT, and 71% of them (*n* = 12) had no residual tumor in LRT specimens. Five patients (13%) were shifted to LRH due to positive surgical margin of trachelectomy (*n* = 4) and positive node (*n* = 1); therefore, LRT was performed in 35 patients (88%). The operative outcomes of LRT are shown in Table 2. The median operative time was 380 min (range: 351–402 min), and the median blood loss was 140 mL (range: 65–255 mL) with no blood transfusion. The residual cervical length was 10 mm (range: 10–12 mm), and 2–0 prorin cerclage was performed in all cases. The median lengths of the parametrium and vagina were 25 mm (range, 21–33 mm) and 21 mm (range, 15–28 mm), respectively. The median length of hospital stay was 15 days (range, 13–19 days), and recovery of voiding function occurred at a median of 14 days. In comparison with type III nerve-sparing LRH (*n* = 30) as a historical cohort, whose patient characteristics are also described in Table 1, neither length of the parametrium, length of the vagina, nor periods of recovery of voiding function differed between the two groups, indicating that the type III nerve-sparing procedure could be accomplished in our LRT. The uterine artery, round ligament, and infundibulopelvic ligament were successfully preserved, and ICG-real-time evaluation revealed preservation of uterine artery blood perfusion in all cases.

In a total of 40 cases, including the five LRH conversion cases, no intraoperative complications were observed; however, two patients (5%) had postoperative complications, namely: internal hernia of the small bowel (*n* = 1), and peritonitis (*n* = 1), wherein reoperation was required in the former case (*n* = 1). 

**Figure 1:**
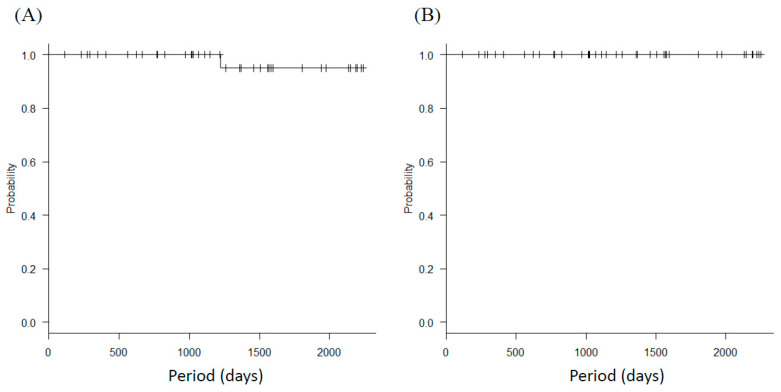
Survival outcomes. Kaplan-Meier analysis of (**A**) disease-free survival (DFS) and (**B**) overall survival (OS) in LRT patients.

The median follow-up period was 41.3 (interquartile range, 25.8–61.3) months; only one patient (3%) experienced recurrence in the para-aortic lymph node region and is currently alive. The 4-year DFS was 95.0% (95% CI: 69.5–99.3%) and the 4-year OS was 100% (Figure 1).

The obstetric outcomes are shown in Table 3. Among the patients who underwent LRT (*n* = 35), nine (26%) attempted pregnancy and seven achieved pregnancy (9 pregnancies), resulting in a pregnancy rate of 76% (7/9). Among these pregnancies, seven were natural conceptions, and two were established by assisted reproductive technology (ART), and eight were delivered by term cesarean section, but one was miscarried during the first trimester. No preterm deliveries were observed, and all babies were appropriate for gestational age. There were no complications during all pregnancies and deliveries. 

## 4. Discussion

Evidence from a recently published prospective randomized trial suggests that oncologic outcomes are inferior when performing minimally invasive radical hysterectomy compared with the open approach in patients with early-stage cervical cancer [5]. Several authors noted the routine use of a uterine manipulator and intracorporeal colpotomy under CO_2_ circulation as two of several possible reasons for the inferior oncologic outcomes associated with minimally invasive approaches. Protective maneuvers of cancer cell spillage are regarded as mandatory to ensure oncologic outcomes [13,20,21,22].

A systematic review including 47 articles on radical trachelectomy reported on 2566 patients and revealed that the positive margin rate of trachelectomy specimens was 3.4% in the vaginal approach, 6.0% in the abdominal approach, and 10.9% in the laparoscopic approach, suggesting that cancer cell spillage into the peritoneal cavity frequently occurred in the laparoscopic approach at trachelectomy [4]. Therefore, we incorporated an extracorporeal trachelectomy technique, in addition to no-look no-touch LRH [13], which we refer to as no-look no-touch LRT. In fact, four cases (10%) resulted in positive trachelectomy margins in our study, which might have caused cancer cell spillage if intracorporeal trachelectomy was conducted. 

In our limited cases of NLNT-LRT, 4-year DFS and OS are good, however, we did not compare the oncologic outcomes between NLNT-LRT and regular LRT. Therefore, the real merit of the no-look no-touch technique is still unknown. A systematic review described that the recurrence rate of regular LRT was 6% (15/238), which was similar to our result, however, it was published before the results of the LACC trial [23]. Recently, a randomized controlled trial of abdominal versus MIS radical trachelectomy incorporated a specific procedure to prevent intraoperative cancer cell spillage into the surgical field. The result of this RCT will disclose the real merit of the no-look no-touch technique [24].

To achieve the goals of adequate radicality and acceptable oncologic outcomes, an adequate free surgical margin at trachelectomy is required; however, the safest distance to maintain between the tumor and the cervical transection remains unclear. Morris et al. reported a case of centropelvic recurrence after radical trachelectomy and concluded that a distance of 5 mm or less is likely to be too limited for radical trachelectomy to be accepted as a treatment for cervical cancer [25]. In contrast, preservation of the cervical stroma is critical for post-trachelectomy pregnancy, and several studies have demonstrated that women receiving radical trachelectomy are at an increased risk of preterm birth [23,26,27,28,29]. Kasuga et al. demonstrated that in pregnancies after abdominal RT, women with a shorter residual cervix were at a higher risk of preterm birth. In particular, women with a residual cervical length < 13 mm in the mid-trimester, which was defined as the distance from the cerclage suture to the external cervical os, were at the highest risk of birth before 34 weeks of gestation. In light of the need to preserve fertility, at least 10 mm of healthy cervical stroma is generally saved in surgical RT procedures [30]. In our study, we secured at least 10 mm residual cervix for acceptable obstetrical outcomes, and a surgical margin of at least 10 mm surgical for better oncologic outcomes. Therefore, we defined “The distance from internal os to the tumor was estimated upon preoperative MRI, to be more than 20 mm” as an inclusion criteria.

In our study, the 4-year PFS and OS were 95% and 100%, respectively, without any multiple recurrences in the pelvis; therefore, we believe our no-look no-touch LRT is oncologically acceptable.

The uterus has three branches of blood supply (ovarian, uterine, and vaginal arteries), and uterine blood flow is a significant factor in uterine viability; however, requiring the preservation of the uterine artery to maintain uterine viability is debatable.

In the female cynomolgus macaque model, Kisu et al. cut the uterus from the vaginal canal to mimic a trachelectomy, in which uterine perfusion was maintained with uterine and ovarian arteries, and found that the uterine artery may be responsible for uterine blood flow to maintain uterine viability [31]. Contrary to this report, Escobar et al. demonstrated that the ovarian vessels may be the primary source of vascularization to maintain uterine viability; however, the authors mainly evaluated the blood perfusion of the uterine fundus by ICG injection [17]. 

Kasuga et al. pointed out that necrotic changes in the cervix might occur after surgery, and that the residual cervix might shorten during pregnancy, which may cause preterm delivery [30]. Thus, blood supply to the residual cervix is also important for better obstetrical outcomes, and we believe that preservation of the uterine artery is essential to maintain better blood perfusion of the residual cervical stroma. However, Tang et al. reported that computed tomography angiography demonstrated that 87.2% of preserved uterine arteries occluded during radical trachelectomy due to traumatic handling of the uterine artery [16].

In our study, we encircled the uterine artery with vessel tape, handled it gently by pulling and pushing the vessel tape, and performed a real-time evaluation of blood perfusion of the uterine artery by ICG fluorescence angiography. Using our method, the uterine artery could be preserved both anatomically and functionally. Our study demonstrated high pregnancy rates and high live birth rates without any preterm deliveries, and we believe these good obstetrical outcomes are owed to our “no-touch” uterine artery preservation technique.

In comparison with type III nerve-sparing LRH, there was no statistical difference in the length of the vagina and parametrium as well as recovery periods to normal bladder function, which meant that our no-look, no-touch LRT can be regarded as a type III nerve-sparing technique. One of the debatable concerns is whether a type III radical procedure is necessary for selected patients in our study. Covens et al. evaluated 842 patients with stage 1A1-1B1 cervical cancer who underwent a radical hysterectomy, and demonstrated that parametrial involvement was associated with larger tumor size, greater depth of myometrial invasion, and pelvic lymph node metastasis. In 536 patients with a tumor size < 2 cm, negative pelvic lymph nodes, and less than 10 mm of cervical stromal invasion, the incidence of parametrial involvement was only 0.6% [32]. In addition, Rob et al. demonstrated acceptable oncologic outcomes and excellent obstetric outcomes by simple trachelectomy without parametrectomy for stage 1b1 cervical cancer without pelvic node metastasis and concluded that simple trachelectomy is a safe and feasible procedure with a high pregnancy rates in early-stage cervical cancer patients [33]. However, data are limited, and more prospective studies are still needed regarding the oncological feasibility of a less radical procedure for trachelectomy.

In conclusion, our results indicated that LRT incorporating specific measures aimed at preventing tumor cell spillage and preserving uterine artery perfusion can be a reliable alternative procedure to regular LRT and ART. However, the study was not a comparison with regular LRT and ART, performed on a limited number of patients, and had short follow up intervals, therefore further studies are needed to validate the utility of no-look no-touch LRT. 

## Figures and Tables

**Table 1:** Clinical characteristics of patients.

	This Study (*n* = 40)	TLRH (*n* = 30)
Age, median (range)	34 (30–37)	44 (33–53)
BMI, median (range), kg/m^2^	20.1 (18.2–21.9)	20.7 (19.5–22.5)
Parity		
Nulliparous	39 (98%)	8 (27%)
Parous	1 (2%)	22 (73%)
Tumor size, median (range), mm	13.5 (8.1–20)	2.1 (1.4–3.1)
Conization prior to LRT, *n* (%)	17 (43%)	4 (13%)
Histology, *n* (%)		
SCC	29 (73%)	14 (47%)
non SCC	11 (27%)	16 (53%)
pT stage, *n* (%)		
1a2	8 (20%)	
1b1	29 (73%)	30 (100%)
2a1	3 (7%)	
pN stage, *n* (%)		
N0	39 (98%)	26 (87%)
N1	1 (2%)	4 (13%)
Adjuvant therapy, *n* (%)		
none	30 (75%)	18 (60%)
chemotherapy	10 (25%)	12 (40%)
Margin status of trachelectomy, *n* (%)		
positive	4 (10%)	
negative	35 (88%)	
Final operation, *n* (%)		
LRT	35 (88%)	
LRH	5 (12%)	

Abbreviations: BMI: body mass index, SCC: squamous cell carcinoma, LRT: laparoscopic radical trachelectomy, and LRH: laparoscopic radical hysterectomy.

**Table 2:** Operative outcomes.

	LRT (*n* = 35) Median (Range)	LRH (*n* = 30) Median (Range)	*p* Value
operation time, min	380 (351–402)		
blood loss, mL	140 (65–255)		
residual cervical length, mm	10 (10–12)		
hospital stay, day	15 (13–19)		
length of parametrium, mm	25 (21–33)	24.7 (10–40)	0.77
length of vagina, mm	21 (15–28)	22.0 (15–37)	0.78
period to recovery residual volume < 50 mL, day	14 (7–60)	20.7 (4–210)	0.6

Abbreviations: LRT: laparoscopic radical trachelectomy, and LRH: laparoscopic radical hysterectomy.

**Table 3:** Obstetrical outcomes.

Patients who attempted pregnancy, *n* (%)	9 (26%)
Pregnancy rate (%)	7/9 (78%)
Gravidity, *n*	9
Parity, *n*	8
Term delivery, *n*	8
Abortion, *n*	1

## Data Availability

The data presented in this study are available on request from the corresponding author.

## Supplementary Materials

The following are available online at https://zenodo.org/record/5509464#.YUH6vH0RVEY, Video S1: supplementary video.

## References

[1] Dargent D., Brun J., Roy M.R.I. (1994). Pregnancies Following Radical Trachelectomy for Invasive Cervical Cancer. Gynecol. Oncol..

[2] Park J.Y., Joo W.D., Chang S.J., Kim D.Y., Kim J.H., Kim Y.M., Kim Y.T., Nam J.H. (2014). Long-Term Outcomes After Fertility-Sparing Laparoscopic Radical Trachelectomy in Young Women with Early-Stage Cervical Cancer: An Asan Gynecologic Cancer Group (AGCG) Study. J. Surg. Oncol..

[3] Kucukmetin A., Biliatis I., Ratnavelu N., Patel A., Cameron I., Ralte A., Naik R. (2014). Laparoscopic Radical Trachelectomy Is an Alternative to Laparotomy with Improved Perioperative Outcomes in Patients with Early-Stage Cervical Cancer. Int. J. Gynecol. Cancer.

[4] Smith E.S., Moon A.S., O’Hanlon R., Leitao M.M., Sonoda Y., Abu-Rustum N.R., Mueller J.J. (2020). Radical Trachelectomy for the Treatment of Early-Stage Cervical Cancer: A Systematic Review. Obstet. Gynecol..

[5] Ramirez P.T., Frumovitz M., Pareja R., Lopez A., Vieira M., Ribeiro R., Buda A., Yan X., Shuzhong Y., Chetty N. (2018). Minimally Invasive Versus Abdominal Radical Hysterectomy for Cervical Cancer. N. Engl. J. Med..

[6] Kanao H., Aoki Y., Takeshima N. (2018). Unexpected Result of Minimally Invasive Surgery for Cervical Cancer. J. Gynecol. Oncol..

[7] Bertagnolli M.M., De Cosse J.J. (1996). Laparoscopic Colon Resection for Cancer—An Unfavorable View. Adv. Surg..

[8] Lee S.W., Gleason N.R., Bessler M., Whelan R.L. (1999). Peritoneal Irrigation with Povidone-Iodine Solution after Laparoscopic-Assisted Splenectomy Significantly Decreases Port-Tumor Recurrence in a Murine Model. Dis. Colon Rectum.

[9] Lee S.W., Southall J., Allendorf J., Bessler M., Whelan R.L. (1998). Traumatic Handling of the Tumor Independent of Pneumoperitoneum Increases Port Site Implantation Rate of Colon Cancer in a Murine Model. Surg. Endosc..

[10] Reymond M.A., Wittekind C., Jung A., Hohenberger W., Kirchner T., Köckerling F. (1997). The Incidence of Port-Site Metastases Might Be Reduced. Surg. Endosc..

[11] Reymond M.A., Schneider C., Kastl S., Hohenberger W., Köckerling F. (1998). The Pathogenesis of Port-Site Recurrences. J. Gastrointest. Surg..

[12] Balli J.E., Franklin M.E., Almeida J.A., Glass J.L., Diaz J.A., Reymond M. (2000). How to Prevent Port-Site Metastases in Laparoscopic Colorectal Surgery. Surg. Endosc..

[13] Kanao H., Matsuo K., Aoki Y., Tanigawa T., Nomura H., Okamoto S., Takeshima N. (2019). Feasibility and Outcome of Total Laparoscopic Radical Hysterectomy with No-Look No-Touch Technique for FIGO IB1 Cervical Cancer. J. Gynecol. Oncol..

[14] Al-Niaimi A.N., Einstein M.H., Perry L., Hartenbach E.M., Kushner D.M. (2011). Uterine Artery Sparing Robotic Radical Trachelectomy (AS-RRT) for Early Cancer of the Cervix. Int. J. Gynaecol. Obstet..

[15] Cibula D., Slama J., Fischerova D. (2008). Update on Abdominal Radical Trachelectomy. Gynecol. Oncol..

[16] Tang J., Li J., Wang S., Zhang D., Wu X. (2014). On What Scale Does It Benefit the Patients If Uterine Arteries Were Preserved During ART?. Gynecol. Oncol..

[17] Escobar P.F., Ramirez P.T., Garcia Ocasio R.E., Pareja R., Zimberg S., Sprague M., Frumovitz M. (2016). Utility of Indocyanine Green (ICG) Intra-Operative Angiography to Determine Uterine Vascular Perfusion at the Time of Radical Trachelectomy. Gynecol. Oncol..

[18] Ebina Y., Mikami M., Nagase S., Tabata T., Kaneuchi M., Tashiro H., Mandai M., Enomoto T., Kobayashi Y., Katabuchi H. (2019). Japan Society of Gynecologic Oncology guidelines 2017 for the treatment of uterine cervical cancer. Int. J. Clin. Oncol..

[19] EZR Installation. www.jichi.ac.jp/saitama-sct/SaitamaHP.files/download.html.

[20] Kohler C., Hertel H., Herrmann J., Marnitz S., Mallmann P., Favero G., Plaikner A., Martus P., Gajda M., Schneider A. (2019). Laparoscopic Radical Hysterectomy with Transvaginal Closure of Vaginal Cuff—A Multicenter Analysis. Int. J. Gynecol. Cancer.

[21] Kanno K., Andou M., Yanai S., Toeda M., Nimura R., Ichikawa F., Teishikata Y., Shirane T., Sakate S., Kihira T. (2019). Long-Term Oncological Outcomes of Minimally Invasive Radical Hysterectomy for Early-Stage Cervical Cancer: A Retrospective, Single-Institutional Study in the Wake of the LACC Trial. J. Obstet. Gynaecol. Res..

[22] Chiva L., Zanagnolo V., Querleu D., Martin-Calvo N., Arévalo-Serrano J., Căpîlna M.E., Fagotti A., Kucukmetin A., Mom C., Chakalova G. (2020). SUCCOR Study: An International European Cohort Observational Study Comparing Minimally Invasive Surgery Versus Open Abdominal Radical Hysterectomy in Patients with Stage IB1 Cervical Cancer. Int. J. Gynecol. Cancer.

[23] Bentivegna E., Gouy S., Maulard A., Chargari C., Leary A., Morice P. (2016). Oncological outcomes after fertility-sparing surgery for cervical cancer: A systematic review. Lancet Oncol..

[24] Chao X., Li L., Wu M., Wu H., Ma S., Tan X., Zhong S., Lang J. (2020). Minimally invasive versus open radical trachelectomy for early-stage cervical cancer: Protocol for a multicenter randomized controlled trial in China. Trials.

[25] Morice P., Dargent D., Haie-Meder C., Duvillard P., Castaigne D. (2004). First Case of a Centropelvic Recurrence After Radical Trachelectomy: Literature Review and Implications for the Preoperative Selection of Patients. Gynecol. Oncol..

[26] Kasuga Y., Nishio H., Miyakoshi K., Sato S., Sugiyama J., Matsumoto T., Tanaka K., Ochiai D., Minegishi K., Hamatani T. (2016). Pregnancy Outcomes After Abdominal Radical Trachelectomy for Early-Stage Cervical Cancer: A 13-Year Experience in a Single Tertiary Care Center. Int. J. Gynecol. Cancer.

[27] Ebisawa K., Takano M., Fukuda M., Fujiwara K., Hada T., Ota Y., Kurotsuchi S., Kanao H., Andou M. (2013). Obstetric Outcomes of Patients Undergoing Total Laparoscopic Radical Trachelectomy for Early Stage Cervical Cancer. Gynecol. Oncol..

[28] Plante M., Gregoire J., Renaud M.C., Roy M. (2011). The Vaginal Radical Trachelectomy: An Update of a Series of 125 Cases and 106 Pregnancies. Gynecol. Oncol..

[29] Rob L., Skapa P., Robova H. (2011). Fertility-Sparing Surgery in Patients with Cervical Cancer. Lancet Oncol..

[30] Kasuga Y., Miyakoshi K., Nishio H., Akiba Y., Otani T., Fukutake M., Ikenoue S., Ochiai D., Matsumoto T., Tanaka K. (2017). Mid-Trimester Residual Cervical Length and the Risk of Preterm Birth in Pregnancies After Abdominal Radical Trachelectomy: A Retrospective Analysis. BJOG.

[31] Kisu I., Banno K., Mihara M., Lin L.Y., Tsuji K., Yanokura M., Hara H., Araki J., Iida T., Abe T. (2012). Indocyanine Green Fluorescence Imaging for Evaluation of Uterine Blood Flow in Cynomolgus Macaque. PLoS ONE.

[32] Covens A., Rosen B., Murphy J., Laframboise S., DePetrillo A.D., Lickrish G., Colgan T., Chapman W., Shaw P. (2002). How Important Is Removal of the Parametrium at Surgery for Carcinoma of the Cervix?. Gynecol. Oncol..

[33] Rob L., Pluta M., Strnad P., Hrehorcak M., Chmel R., Skapa P., Robova H. (2008). A Less Radical Treatment Option to the Fertility-Sparing Radical Trachelectomy in Patients with Stage I Cervical Cancer. Gynecol. Oncol..

